# Evaluation of plasma calprotectin as a marker for infection in various clinical settings: a prospective observational study

**DOI:** 10.1186/s40635-026-00923-3

**Published:** 2026-05-29

**Authors:** Tobias Zimmermann, Timothy Arthur Chandos Snow, Pedro Lopez-Ayala, Ahmed Al-Hindawi, Samer Elkhodair, Nishkantha Arulkumaran, Martin Siegemund, Mervyn Singer, David Brealey

**Affiliations:** 1 Bloomsbury Institute of Intensive Care Medicine, Centre for Respiratory Medicine, University College London, Gower St, London, WC1E 6BT UK; 2 Intensive Care Unit, Department of Acute Medicine, University Hospital Basel, University of Basel, Basel, Switzerland; 3 Cardiovascular Research Institute Basel, University Hospital Basel, University of Basel, Basel, Switzerland; 4 Emergency Department, University College London Hospital, London, UK

**Keywords:** Infection, Biomarker, Diagnosis, Sepsis, Calprotectin, Inflammation

## Abstract

**Background:**

Reliably distinguishing infection from sterile inflammation is a major clinical challenge. Uncertainty can lead to unnecessary courses of antibiotics, fueling antimicrobial resistance and adverse effects. Calprotectin, a biomarker released by activated immune cells, may inform decision-making.

**Methods:**

This prospective, observational, single-centre study recruited patients with suspected infection who provided blood samples on enrolment from the Emergency Department (ED) and Intensive Care Unit (ICU) of a central London university hospital. A separate longitudinal study with five days’ blood sampling was performed in patients undergoing elective major non-cardiac surgery, in whom infection was adjudicated according to the Standardised Endpoints in Perioperative Medicine (StEP) initiative. Diagnostic adjudication was performed blinded to calprotectin. The primary outcome was the ability of calprotectin to diagnose infection. Secondary outcomes included a comparison to C-reactive protein (CRP).

**Results:**

427 patients were included, of whom 186 (44%) were female. Of 245 ED patients, 71 (29%) had active cancer and 56 (23%) were on immunosuppressants. The median calprotectin level in the no-infection group was 1.97 mg/L (IQR 1.02–3.39), compared to 2.76 (IQR 1.65–5.08) mg/L in low-probability infection, 2.63 mg/L (IQR 1.83–5.23) in high-probability infection, and 2.64 mg/L (IQR 1.49–4.45) in patients with confirmed infection. Ordinal regression analysis found no meaningful association between calprotectin levels and infection, or bacterial infection. Logistic regression showed an unadjusted AUC of 0.53 (95%-CI 0.45–0.62) for calprotectin and a binary outcome of infection compared to an AUC of 0.63 (95%-CI 0.55–0.71) for CRP. Similar results were seen in a sensitivity analysis excluding patients with cancer or immunosuppression. In 98 ICU patients, neither calprotectin nor CRP showed a meaningful association with an adjudicated diagnosis of infection or ICU death. In the peri-operative cohort, calprotectin levels remained elevated over 5 days, but with no difference between patients developing or not developing infection.

**Conclusion:**

Calprotectin showed only limited ability to differentiate infection from inflammation across ED, ICU, and elective surgery patients. Excluding patients with cancer or immunosuppression did not alter the overall findings.

**Supplementary Information:**

The online version contains supplementary material available at 10.1186/s40635-026-00923-3.

## Key points

**Question**

Can calprotectin reliably differentiate infection from sterile inflammation in different clinical settings and aid physicians in their decision making?

**Findings**

In this prospective, observational, single-centre study recruiting patients suspected to have infection including a substantial proportion of especially vulnerable patients with cancer or immunosuppression from the ED, ICU and a peri-operative setting, no meaningful association was found between Calprotectin and infection. Excluding patients with cancer or immunosuppression did not alter the overall findings.

**Meaning**

Calprotectin showed only limited ability to differentiate infection from inflammation across ED, ICU, and elective surgery patients.

**Supplementary Information:**

The online version contains supplementary material available at 10.1186/s40635-026-00923-3.

## Introduction

Infection and sepsis are common causes of emergency department (ED) visits, hospital admission and the need for intensive care [1]. Timely identification and initiation of treatment are key to avoiding high rates of morbidity and mortality [2]. However, distinguishing between infectious and non-infectious causes of inflammation can be challenging, especially in patients with a strong systemic inflammatory response, such as following surgery or trauma. The fear of severe consequences through delay, in combination with the long turnover time for a positive microbiology culture result, leads to unnecessary prescription of antibiotics, promoting antimicrobial resistance and adverse drug reactions [3–5]. Biomarkers such as C-reactive protein (CRP), procalcitonin (PCT) and white cell count (WCC) are often used on a daily basis, despite limited evidence for their ability to reliably distinguish infection from sterile inflammation [6–8].

To address this unmet clinical need, researchers have been searching for superior biomarkers, both rule-in and rule-out, but none as yet has been widely established [9–11]. Omics and multiplex protein approaches show promise compared to single-biomarker strategies, but are currently limited in routine clinical practice, mainly due to technical and financial reasons [8, 12, 13].

Calprotectin, an antimicrobial protein and heterodimer of the S100 calcium binding proteins A8 and A9 [14], has favourable kinetics since it is released from the cytosol of activated immune cells such as neutrophils, macrophages and monocytes unlike, for example, CRP which is synthesised and released from the liver [15]. Its biological functions include direct antimicrobial actions via binding of zinc and manganese, and pro-inflammatory signalling as a damage-associated pattern (DAMP) protein with cytokine-like effects [16]. Calprotectin is used as a stool biomarker for inflammatory bowel disease [17, 18], and has recently garnered interest as a potential new infection biomarker, however its clinical value in this area remains largely unknown [19].

We thus aimed to investigate the utility of plasma calprotectin across different patient populations with a high prevalence of cancer and immunosuppression from the ED, the ICU and the peri-operative medicine.

## Methods

### Study design, setting and population

This prospective observational single-centre cohort study was conducted at University College Hospital, London, United Kingdom between September 2021 and November 2022. Written informed patient or consultee consent/agreement was obtained from all patients. Since University College London Hospital offers a wide variety of cancer services, we expected a larger-than-average representation of an especially vulnerable population of patients suffering from active cancer and immunosuppression in our study cohorts.

Adult patients 18 years and over presenting to the ED or admitted to the ICU were screened from Monday to Sunday during working hours. ED and ICU patients were subsequently approached for enrolment into the study if there was a suspicion of infection by the clinical team and blood cultures were being taken. Recruitment and sampling of ICU patients were independent of ICU length of stay at the time of screening but strictly connected to the time of clinical suspicion of infection. With this, we aimed to recruit patients and sample blood as soon as signs of infection became clinically apparent. In the ICU this sometimes occurred days post-admission.

Patients undergoing major elective, non-cardiac surgery were identified electronically before their procedure and approached for participation.

Exclusion criteria included palliative treatment intent, severe anaemia (e.g. due to hyper-haemolysis), and any contraindication to blood transfusion (e.g. religious reasons, or multiple antibodies). Only patients with at least one valid measurement of calprotectin and CRP were included in the analysis.

The study and study protocol were approved by the London—Queen Square Research Ethics Committee 20/LO/1024 Medical Research Ethics Committee (IRAS 266594). The study was conducted according to the principles of the Declaration of Helsinki. Reporting is in accordance with STARD guidelines.

### Clinical management and diagnostic adjudication

This study had no impact on clinical management of included patients as samples were collected and stored for subsequent batch processing and analysis.

For ED and ICU patients, a post hoc expert panel (TZ, DB, MSin) performed a diagnostic adjudication after patient discharge. Adjudication was conducted by manually reviewing available electronic patient records including medical history, presenting symptoms, vital signs, imaging, and microbiology/virology results in groups of two experts. After patient data review, in-depth case discussion followed to determine the likelihood of infection (no infection, low probability, high probability, confirmed infection) [20]. In cases of disagreement or high uncertainty, the third adjudicator arbitrated the final diagnosis. Since adjudication was performed through case discussion, interrater agreement was not formally assessed. For ED patients, CRP and WCC were excluded from the adjudication process to allow a non-biased comparison between CRP and calprotectin. At the time of study, procalcitonin was not measured. A diagnosis of definite bacterial infection required a clinical picture in line with infection and a positive bacterial culture or polymerase chain reaction (PCR) result from blood, sputum, or other samples taken from areas of suspected infection (e.g. wounds, synovial fluid). Positive culture results were reviewed for likely contamination or colonisation.

In patients undergoing major elective surgery, the Standardised Endpoints in peri-operative Medicine (StEP) criteria were used to adjudicate for presence of infection [21].

### Blood sampling and laboratory methods

Study blood samples from ED and ICU patients were taken together with the collection of blood cultures. ED patients had only one sample taken. For ICU patients, a second sample was drawn 1–2 days after the first sample and a third sample taken 5–7 days after the first. Patients undergoing major surgery had the first sample taken prior to surgery. A postoperative sample was obtained on the day of the surgery following return from the operating theatre. A third sample was taken 1–2 days after surgery and a fourth sample 5–7 days after surgery.

All samples were collected into lithium-heparin vacutainers, centrifuged within 4 h, aliquoted, and frozen for batch analysis. Routine blood tests, including CRP, were performed from fresh blood in the hospital laboratory at UCLH. Plasma calprotectin measurements were performed from frozen samples at the Gentian Laboratories in Norway using a Particle-Enhanced Turbidimetric Immunoassay (GCAL^®^ Calprotectin Immunoassay Gentian Diagnostics, Moss, Norway). The assay’s limit of quantification in lithium-heparin samples was 0.31 mg/L, with an upper reference limit (URL) of 0.97 mg/L. A previous study investigating (pre-) analytical factors of calprotectin measurements showed relevant differences in absolute values based on sample type and handling [22]. Comparison of calprotectin concentrations between different studies should be made with this in mind. All laboratory measurements were conducted blinded to clinical data.

### Outcomes and sensitivity analyses

The primary ordinal outcome was an adjudicated diagnosis of infection (no infection, low probability, high probability, and confirmed infection). Where a binary outcome was required, patients in the no-infection and low-probability groups were combined as unlikely infection, and patients in the high probability and confirmed infection groups as likely infection. For the ED group, a secondary outcome was bacterial infection. Pre-defined subgroup analyses were performed across various patient characteristics; as these were exploratory, no significance testing was performed.

Due to the high number of patients in our cohorts with active cancer or immunosuppression, and the potential impact of these conditions on the performance of the biomarkers, a sensitivity analysis was performed excluding those patients. To investigate potential dependence, correlation analyses between calprotectin, CRP and neutrophil counts were performed.

### Data management and statistical methods

Data from hospital medical records were collected and managed using REDCap electronic data capture tools hosted on-site [23]. Continuous variables are presented as median with interquartile range (IQR). Categorical variables are expressed as counts and proportions. Wilcoxon and Kruskal–Wallis tests were applied for comparisons between continuous variables. Pearson’s Chi-squared test without continuity correction was used for comparisons of categorical variables. The Spearman correlation coefficient was classified as (low: 0–0.3, moderate 0.3–0.7, high 0.7–1).

To assess the association between biomarkers and outcome, multivariable regression models were used, including proportional odds regression for ordinal outcomes and logistic regression for binary outcomes. Restricted cubic splines were used to model continuous variables to avoid both dichotomisation and imposing linearity [24]. Models were adjusted for potential confounders selected based on clinical knowledge and previous literature, independent of p-value, and included age, sex, active cancer disease, ongoing medical immunosuppression and, for the ED cohort, the highest recorded National Early Warning Score (NEWS-2). Interactions between biomarkers and immunosuppression and active cancer disease were tested using ANOVA. To assess discrimination of the biomarkers for binary outcomes, the area under the receiver operating characteristic curve (AUC) was calculated. The magnitude of the effect of the biomarkers was assessed graphically via dose–response plots. Reference levels for calprotectin and CRP were set to the URL according to the manufacturers’ information (0.97 mg/L for calprotectin, 10 mg/L for CRP), and used as a reference for calculating odds ratios.

Cut-offs for rule-in and rule-out were investigated based on a binary outcome. Targets were maximising positive predictive value (PPV) and negative predictive value (NPV) for rule-in and rule-out, respectively. PPV/specificity (true negative rate, TNR) and NPV/sensitivity (true positive rate, TPR) plots were created to visualise performance of possible cut-offs.

Due to a relatively low number of missing values, full-case analysis was performed. No specific sample size calculation was performed for this observational diagnostic study, due to high levels of uncertainty about how the biomarker would perform in this special cohort and lack of reliable performance metrics to base a calculation on. All hypothesis testing was two-tailed, with p-values < 0.05 considered statistically significant. All statistical analyses were performed using R Statistical Software (v4.4.1; R Core Team (2024)) [25].

### Role of the funding source

This study was sponsored and funded by Gentian AS, Norway. Gentian AS had no role in the design or conduct of the study, data analysis and interpretation as well as drafting and publishing this manuscript. Results of the analyses were presented to the company before publication.

## Results

### Emergency department

Two hundred and forty-five of 253 patients recruited from the ED were eligible for analysis (Supplementary Fig. 1). Median age was 55 years (IQR 32–70) and 118 (48%) were female (Table 1). Seventy-one patients (29%) had active cancer disease and 56 (23%) were receiving immunosuppressants. Antibiotic therapy was started in 190 patients (78%), and 157 (64%) were admitted to hospital. Blood cultures taken on the ED yielded a positive result in 15 (6%) of all cases. The overall median plasma calprotectin concentration was 2.5 mg/L (IQR 1.6–4.7), with no significant difference between women (2.3 mg/L, IQR 1.6–4.1) and men (2.8 mg/L, IQR 1.6–5.5, *p* = 0.20). Calprotectin levels showed similar distribution across different suspected sites of infection (*p* = 0.340) (Supplementary Fig. 2).

**Table 1:** Demographics of emergency department (ED), intensive care unit (ICU) and elective surgical patients

	ED	Surgery	ICU
	*N* = 245^*1*^	*N* = 84^*1*^	*N* = 98^*1*^
Characteristics			
Sex (female)	115 (48%)	36 (43%)	35 (36%)
Age	55 (32, 70)	65 (55, 71)	58 (46, 70)
NEWS-2	3 (2, 5)	–	–
SOFA score	–	–	6 (4, 9)
Missing	–	–	1
Adjudicated diagnosis			
No infection	30 (12%)	50 (60%)	32 (33%)
Low probability	34 (14%)	0 (0%)	32 (33%)
High probability	91 (37%)	0 (0%)	13 (13%)
Confirmed infection	90 (37%)	34 (40%)	21 (21%)
Bacterial infection	103 (42%)	–	–
Laboratory tests			
Calprotectin	2.5 (1.6, 4.7)	2.08 (1.29, 3.39)	3 (2, 6)
Missing	–	6	3
White cell count	9.7 (6.4, 13.9)	11.9 (9.6, 14.6)	10 (7, 17)
Missing	1	14	1
C-reactive protein	52 (15, 122)	50 (26, 71)	146 (63, 218)
Missing	2	22	5
Creatinine	83 (66, 104)	68 (56, 82)	75 (56, 111)
Missing	2	13	3
Comorbidities			
Active cancer	71 (29%)	74 (88%)	32 (33%)
Inflammatory bowel disease	9 (3.7%)	2 (2.4%)	1 (1.0%)
Vasculitis	1 (0.4%)	0 (0%)	0 (0%)
Auto-immune disease (e.g. SLE, rheumatoid arthritis)	13 (5.3%)	3 (3.6%)	4 (4.1%)
Chronic pancreatitis	1 (0.4%)	0 (0%)	2 (2.0%)
Known chronic infection (e.g. tuberculosis)	4 (1.6%)	0 (0%)	0 (0%)
Trauma (on this admission)	1 (0.4%)	0 (0%)	0 (0%)
HIV	2 (0.8%)	0 (0%)	3 (3.1%)
Liver cirrhosis	2 (0.8%)	1 (1.2%)	4 (4.1%)
Dialysis dependence	0 (0%)	0 (0%)	2 (2.0%)
Bone marrow transplant	8 (3.3%)	0 (0%)	5 (5.1%)
Immunosuppressants			
Systemic steroids	20 (8.2%)	2 (2.4%)	6 (6.1%)
Methotrexate	2 (0.8%)	0 (0%)	1 (1.0%)
Chemotherapy to manage cancer	25 (10%)	19 (23%)	5 (5.1%)
Monoclonals (e.g. Infliximab)	9 (3.7%)	1 (1.2%)	3 (3.1%)

^*1*^n (%); median (Q1, Q3)

*NEWS-2* National Early Warning Score Version 2, *SOFA* Sequential Organ Failure Assessment Score, *SLE* systemic lupus erythematosus, *HIV* human immunodeficiency virus

**Fig. 1:**
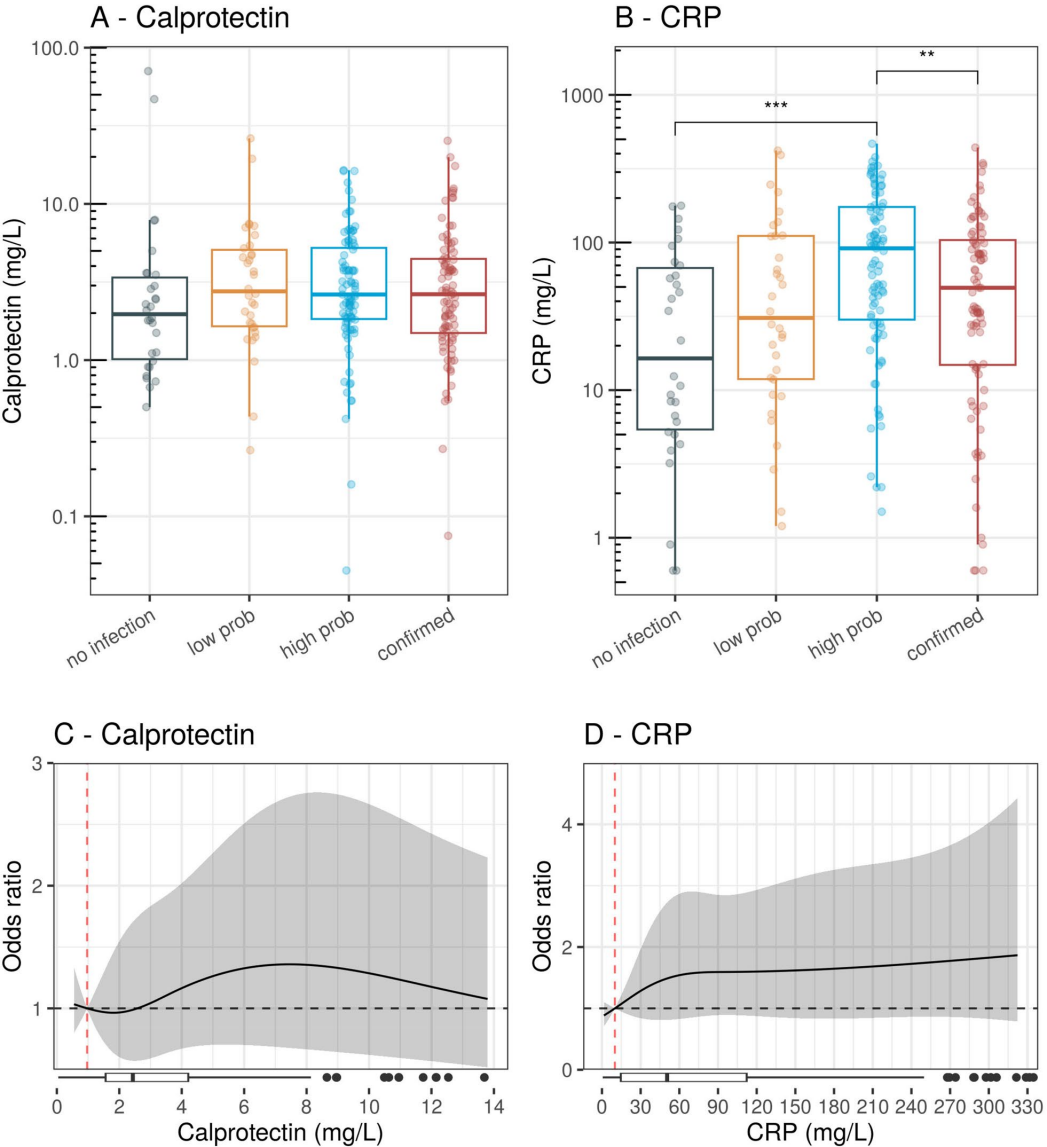
Calprotectin (**A**) and C-reactive protein [CRP] (**B**) concentrations on a log10 scale, in the emergency department cohort, stratified by the four adjudicated diagnoses: no infection, low probability, high probability, and confirmed infection. Only significant differences between groups are displayed. Dose–response plots show the level-dependent association of calprotectin (**C**) and CRP (**D**) with the diagnosis of infection. The horizontal boxplot at the bottom displays the distribution of the biomarker data. Horizontal dashed black line: odds ratio of 1. Vertical red dashed line: reference levels of the biomarkers (calprotectin 0.97 mg/L, CRP 10 mg/L). While dose–response plots were generated using all available data, only 95% of the data are displayed for better visualisation. prob: probability

Among eligible ED patients, 30 (12%) had an adjudicated diagnosis of no infection, 34 (14%) low probability, 91 (37%) high probability, and 90 (37%) confirmed infection. The median calprotectin level in the no-infection group was 1.97 mg/L (IQR 1.02–3.39), compared to 2.76 mg/L (IQR 1.65–5.08) for low probability, 2.63 mg/L (IQR 1.83–5.23) for high probability, and 2.64 mg/L (IQR 1.49–4.45) for confirmed infection (Fig. 1A). CRP concentrations in these four groups were 17.05 mg/L (IQR 5.43–67.35), 31.00 mg/L (IQR 11.88–110.85), 91.30 mg/L (IQR 30.05–174.15), and 49.35 mg/L (IQR 14.83–103.90), respectively (Fig. 1B).

Ordinal regression analysis revealed no meaningful association between either calprotectin or CRP and the outcome (Fig. 1C, D). The chunk tests for interaction between calprotectin and immunosuppression/active cancer disease were non-significant (p = 0.330) and therefore excluded from further analysis.

**Fig. 2:**
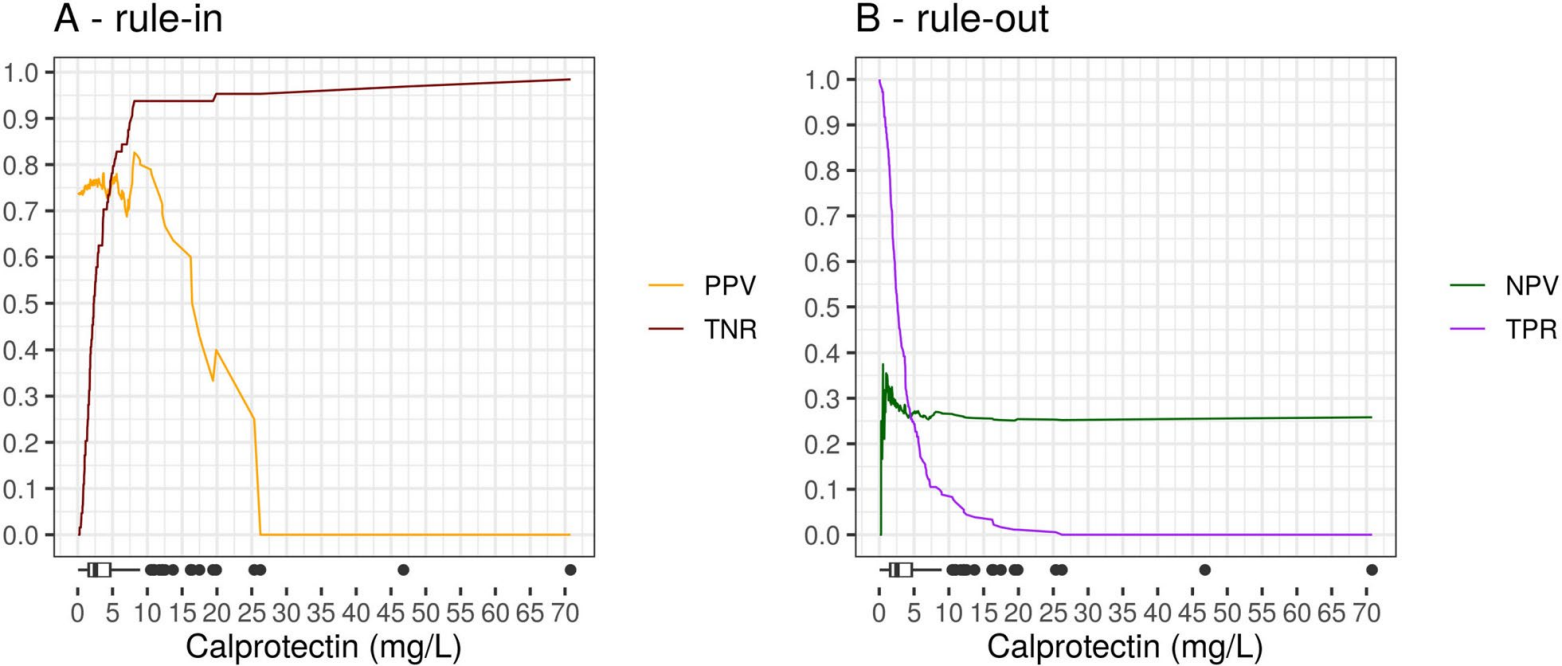
Continuous display of calprotectin cut-off thresholds and their performance metrics for rule-in (**A**) and rule-out (**B**) in the Emergency Department cohort. Boxplots at the bottom show the distribution of patients. *PPV* positive predictive value. *TNR* true negative rate (sensitivity). *NPV* negative predictive value. *TPR* true positive rate (specificity)

For rule-in, a calprotectin cut-off of 8.15 mg/L delivered the maximum PPV of 83% with specificity of 94% and sensitivity of 11% (Fig. 2A). For rule-out, the cut-off for a maximum NPV of 37% was 0.55 mg/L with sensitivity 97% and specificity 5% (Fig. 2B).

**Fig. 3:**
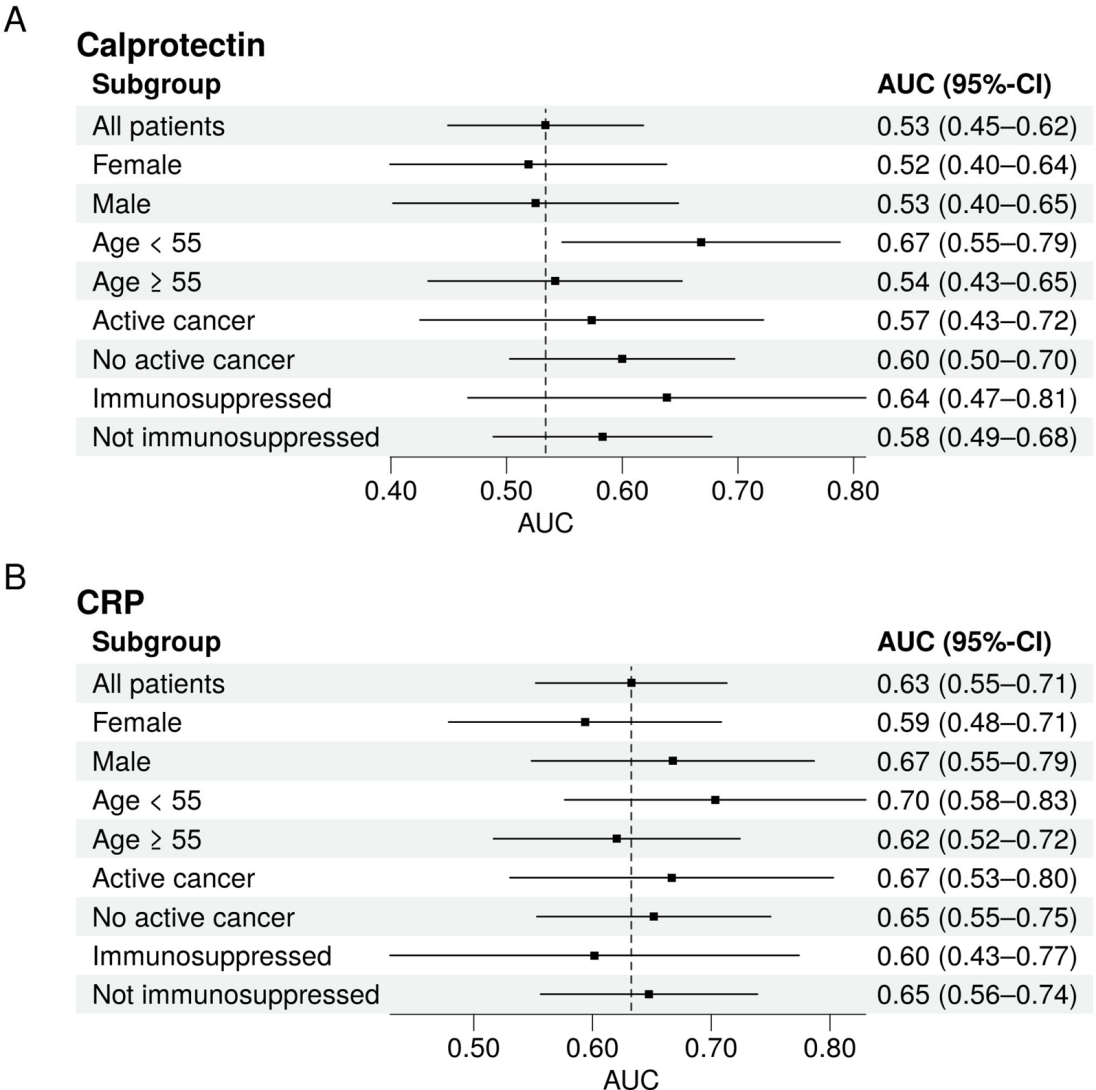
Exploratory analysis of calprotectin (**A**) and CRP (**B**) performance in predefined subgroups of the emergency department cohort. *AUC* area under the receiver operating characteristic curve. *CI* confidence interval

Exploratory analysis of predefined subgroups revealed an overall consistent performance of both biomarkers with the only exception that calprotectin showed improved performance in younger patients (Fig. 3).

Sensitivity analysis excluding 86 patients (35%) who had active cancer and/or receipt of immunosuppressant therapy, revealed similar results to the main cohort (Supplementary Fig. 3).

**Fig. 4:**
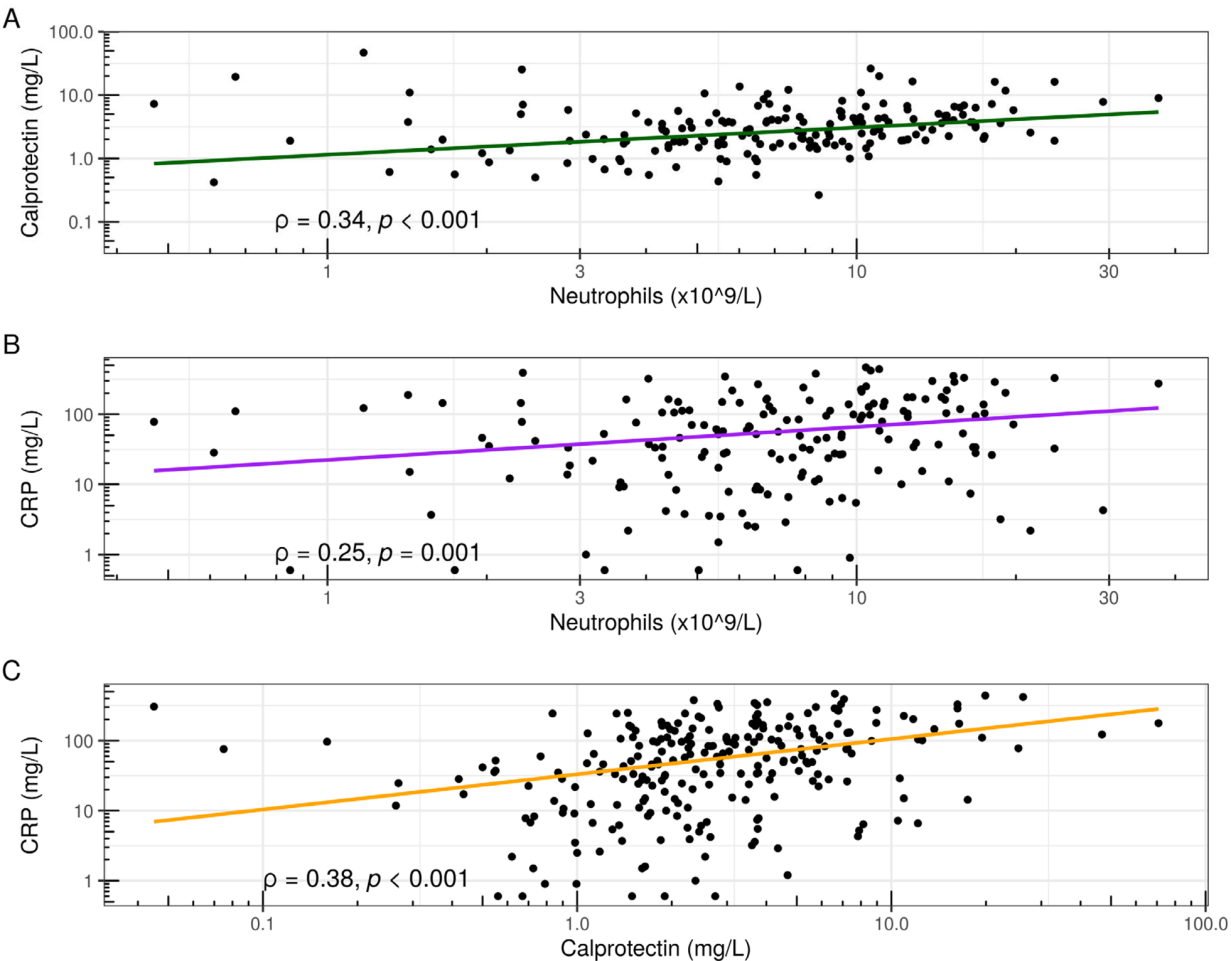
Correlation analysis between calprotectin (**A**) and CRP (**B**) and neutrophil levels, and between calprotectin and CRP (**C**) in the emergency department cohort. Both axes are on a logarithmic scale

Correlation analysis showed a moderate correlation between calprotectin levels and neutrophil counts (Spearman rho 0.34, *p* < 0.001), and a lower correlation between CRP and neutrophils (Spearman rho 0.25, *p* = 0.001) (Fig. 4A, B). Calprotectin and CRP levels had a moderate correlation (Spearman rho 0.38, *p* < 0.001) (Fig. 4C).

For the secondary outcome of an adjudicated diagnosis of bacterial infection, calprotectin levels were higher in bacterial infection compared to non-bacterial infection; however, the difference did not reach statistical significance (3.01 mg/L, IQR 1.85–5.26 vs 2.27 mg/L, IQR 1.49–3.88, *p* = 0.063). CRP levels were higher in bacterial infection (96 mg/L, IQR 37.10–168.85 vs 33.65 mg/L, IQR 11.00–85.78, *p* < 0.001) (Supplementary Fig. 4A, B). Logistic regression showed a near-linear significant association between CRP and bacterial infection, and only a less clear association with calprotectin (Supplementary Fig. 4C, D). The unadjusted AUC for bacterial infection was 0.57 (95%-CI 0.50–0.65) for calprotectin and 0.70 (95%-CI 0.63–0.76) for CRP (*p* = 0.002).

### Intensive care unit

**Fig. 5:**
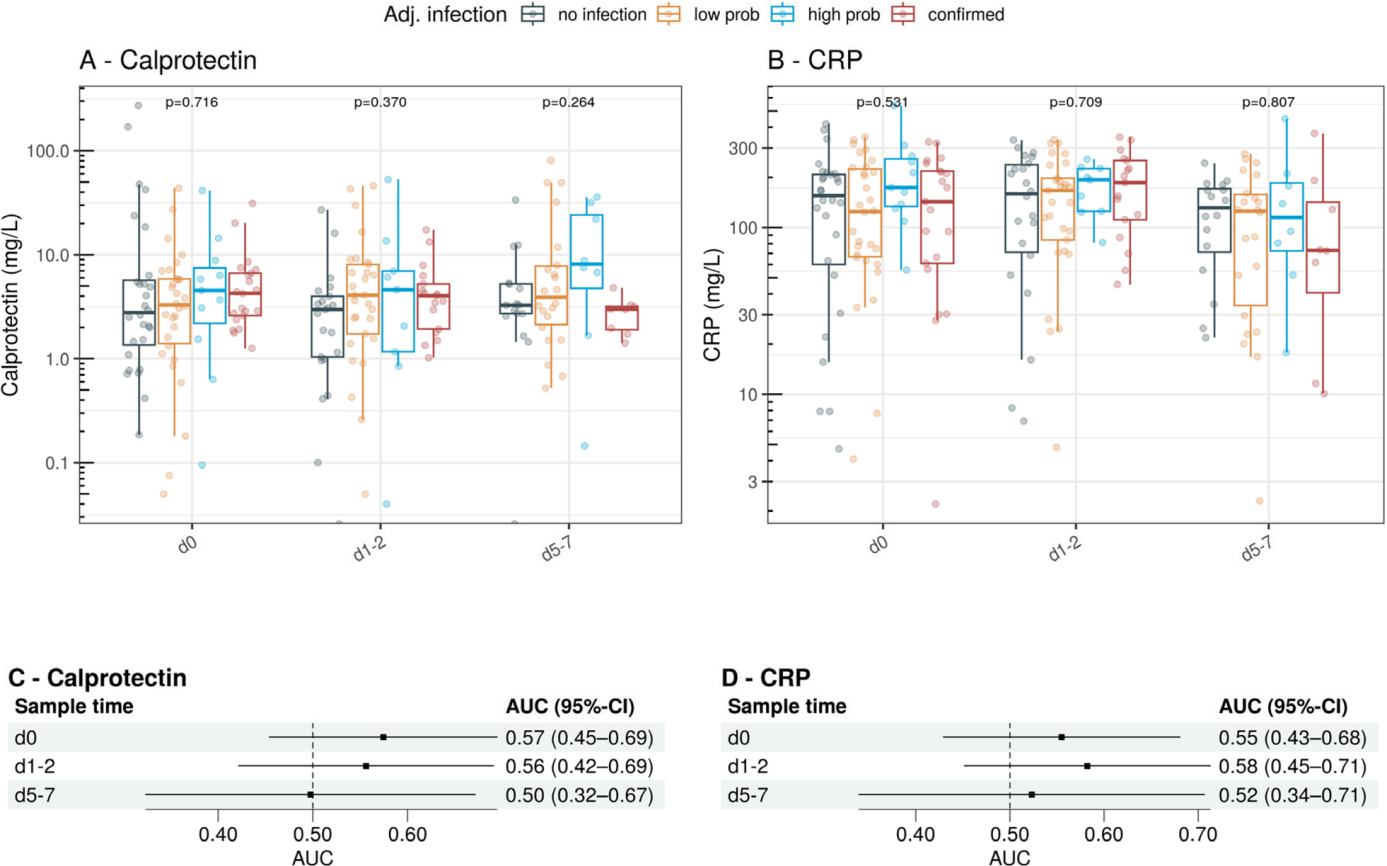
Calprotectin (**A**) and CRP (**B**) concentrations on a logarithmic scale, in the intensive care unit cohort, for the different sample time points, and stratified by the four adjudicated diagnoses: no infection, low probability, high probability, and confirmed infection. Forest plots show the discriminatory ability of calprotectin (**C**) and CRP (**D**) for a binary outcome of likely infection and unlikely infection at the various sampling time points. *Prob* probability. *AUC* area under the curve

In a cohort of 98 ICU patients, with a median age of 58 years (IQR 46–70), 35 (36%) were women, 55 (56%) required mechanical ventilation, and 33 (34%) required at least one vasopressor or inotrope. A diagnosis of high probability or confirmed infection was adjudicated in 34 patients (34%). The median calprotectin level in the first sample was 2.78 mg/L (IQR 1.36–5.69) in the no-infection group and 3.29 (IQR 1.40–5.85) in the low-probability group, compared to 4.53 mg/L (IQR 2.32–7.56) in the high-probability group and 4.25 mg/L (IQR 2.60–6.64) in the confirmed infection group. Across the different sample time points, there was no notable difference between the adjudicated diagnoses for both calprotectin and CRP (Fig. 5A, B). Likewise, diagnostic discrimination was comparable for both biomarkers (Fig. 5C, D). There was no significant correlation between calprotectin levels and total Sequential Organ Failure Assessment (SOFA) scores in ICU patients at any of the sample time points (all *p* > 0.05) (Supplementary Fig. 5).

### Major non-cardiac surgery

Among 84 patients undergoing elective major non-cardiac surgery, median age was 65 years (IQR 55–71) and 36 (43%) were women. A diagnosis of active cancer was present in 74 (88%) patients.

**Fig. 6:**
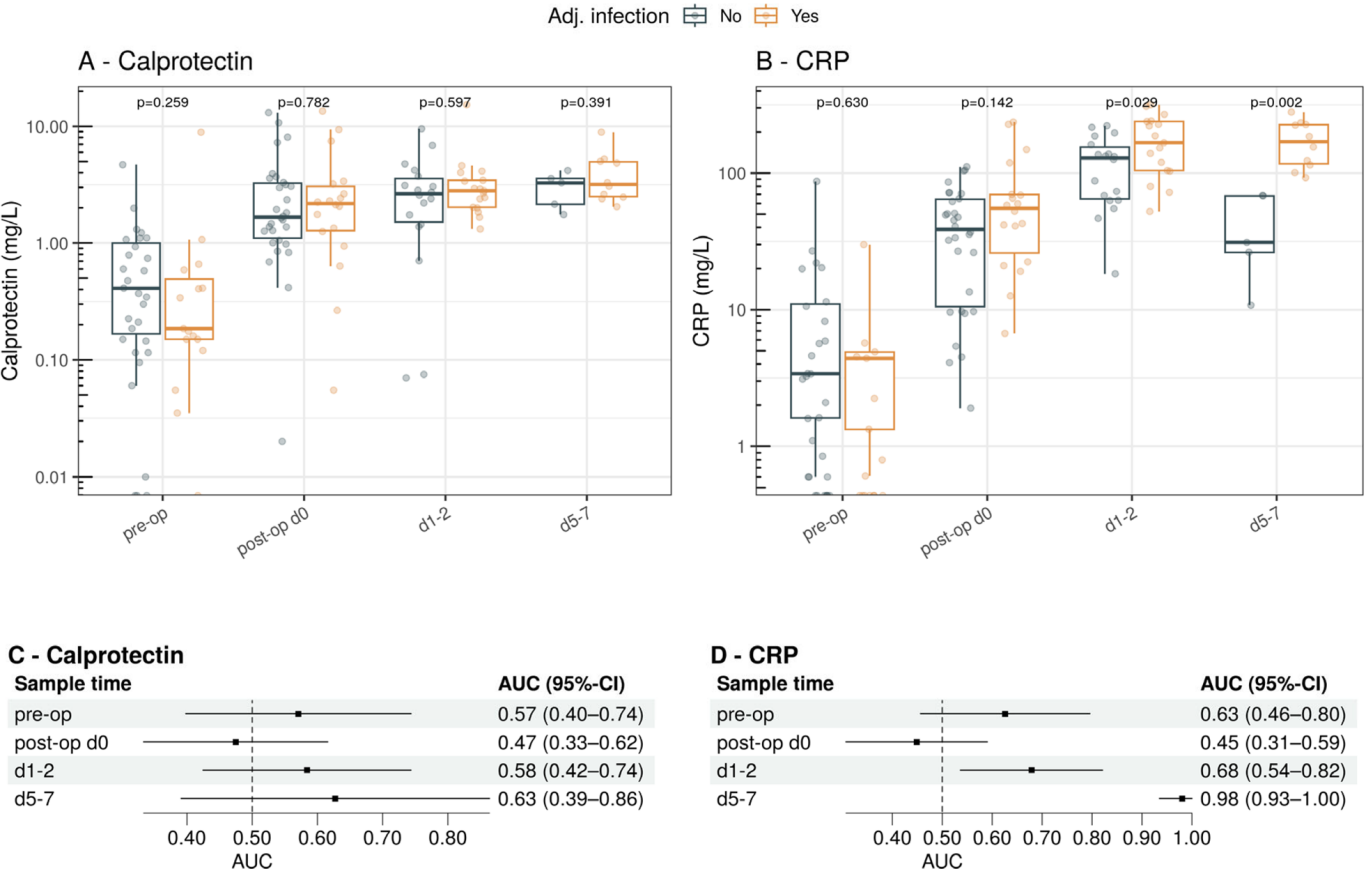
Calprotectin (**A**) and CRP (**B**) concentrations on a logarithmic scale, in the surgical cohort, for the different sample time points and stratified by infection. Forest plots show the discriminatory ability of calprotectin (**C**) and CRP (**D**) for a binary outcome of likely infection and unlikely infection at the various sampling time points. *AUC* area under the curve

Pre-operative calprotectin concentrations were low (0.3 mg/L, IQR 0.1–0.8), and below the URL (0.97 mg/L). Calprotectin concentrations were elevated when measured post-operatively on the day of surgery (2.08 mg/L, IQR 1.30–3.38), on postoperative days 1–3 (2.57 mg/L, IQR 1.86–3.59) and days 5–7 (3.29 mg/L, IQR 2.15–4.38). No difference was found between patients who developed postoperative infection or not (Fig. 6A, B). By comparison, significantly higher CRP levels were measured in patients with infection at days 1–2 and 5–7. This was also reflected in the increased diagnostic discrimination at these time points (Fig. 6C, D).

## Discussion

This prospective, observational study investigated plasma calprotectin as a diagnostic biomarker for infection in patients presenting to the ED, admitted to the ICU, and/or undergoing major elective non-cardiac surgery. Calprotectin levels were elevated in patients suspected to have infection and after surgery; however, no meaningful association with an adjudicated diagnosis of infection was found across all three cohorts.

The findings of our study extend and corroborate previous studies investigating calprotectin as a marker for infection or sepsis [26–29]. The prospective CASCADE study recruited 395 patients from a German ED to evaluate calprotectin for an adjudicated diagnosis of bacterial infection and sepsis [30]. They found high diagnostic discrimination for both outcomes (AUC 0.90, 95%-CI 0.86–0.93 and AUC 0.83, 95%-CI 0.78–0.88, respectively). One limitation of their analysis lies in the choice of control group—adults without signs of acute infection—which explains the differences observed between their findings and those of this study and others.

A retrospective analysis of blood samples of 4732 patients included in the Swecrit biobank study found an AUC of 0.61 (95%-CI 0.59–0.62) for calprotectin and 0.72 (95%-CI 0.71–0.73) for CRP for detecting confirmed sepsis at ICU admission [31]. This study also derived a calprotectin cut-off of 1.6 mg/L for sepsis based on the Youden index, resulting in a sensitivity of 59% (95%-CI 57–61) and specificity of 57% (95%-CI 55–59). Determination of a single cut-off based on the Youden index, however, disregards the clinical purpose of an infection biomarker. In our study, we investigated two cut-offs for calprotectin in the ED cohort: one for rule-in (maximising PPV) and one for rule-out (maximising NPV). Both derived cut-offs are not suitable for clinical use due to the limited performance of the biomarker; applying the derived rule-in cut-off to our cohort reveals only 19 true positives (sensitivity 10%), while applying the rule-out cut-off leads to only 3 true negatives (specificity 5%).

This present study is, to our knowledge, the first to specifically investigate the role of calprotectin in patients with cancer and on medical immunosuppressive medication. Adjusted regression analysis revealed no significant interaction in both subgroups of patients. A moderate correlation was found between calprotectin levels and neutrophil counts which corroborates a previous study that reported lower calprotectin levels in neutropenia [31].

Ours is also the first study to evaluate calprotectin in a peri-operative setting. While calprotectin levels are routinely elevated after surgery, they provided no reliable discrimination between postoperative inflammation and infection. Calprotectin is released from activated immune cells such as neutrophils. As these cells are also activated by non-infectious causes of inflammation, such as trauma during surgery, it is perhaps understandable how no strong discriminative signal could emerge.

In the Swecrit study, fungal sepsis had the highest median level of calprotectin at around 3.0 mg/L while bacteraemic sepsis had higher levels compared to non-bacteraemic sepsis (2.6 mg/L and 1.9 mg/L, respectively). This might hint towards a potential use of calprotectin to differentiate between different types of infection rather than differentiating infection from inflammation.

In our ICU cohort, we found no difference in calprotectin levels at any sample time point between patients with and without infection. Since suspicion of infection by the clinical team was a trigger for recruitment, it is ultimately unclear at which point of the infection/sepsis course of disease samples were taken. It is likely, however, that in those patients in whom high levels of inflammation are often present and mixed with signals from an infectious aetiology, single-biomarker strategies may not improve management of these patients. Omics or multiplex approaches are more likely to provide additional insights in these patients; however, these carry their own risks and limitations [8].

In summary, based on the findings of this and previous studies, calprotectin does not seem to be able to reliably discriminate infection from inflammation and has no advantage over already established markers such as CRP as a general infection marker. Future research around calprotectin should perhaps focus on its potentially improved performance in younger patients and its characteristic of early release from immune cells, unlike CRP which has to first be synthesised and released by the liver. No clinical study evaluating calprotectin so far has had the time resolution necessary to properly investigate this potential advantage of calprotectin over other biomarkers.

This study has several strengths and limitations. Definitions of infection vary between studies and no widely accepted and validated criteria exist. For our study, post hoc expert diagnostic adjudication for infection was performed in all patients included in this study, and was blinded to calprotectin and CRP results for the ED cohort. Despite rigorous methodology and best efforts, some patients may have been misclassified, potentially influencing performance characteristics. It is however unlikely that misclassification of a few patients would have meaningfully changed the main results of this study. This was a prospective single-centre observational study recruiting patients at a hospital with a clinical focus on cancer medicine, haematological diseases and elective major non-cardiac surgery. Generalisability of the results to other patient populations is unclear, although our statistical analysis did not reveal an interaction between cancer or immunosuppression and calprotectin levels.

## Conclusion

This study found only limited ability of calprotectin to discriminate infection from sterile inflammation, and no superiority compared to CRP in various clinical settings. Future studies should focus on the potentially unique early release characteristic of calprotectin which may offer value in the early detection of the inflammatory host response to infection.

## Supplementary Information

Supplementary Material 1.

## Data Availability

Data collected in the course of this study are protected and not available publicly. Reasonable inquiries for access to portions of the coded data can be directed to the corresponding author.

## References

[1] Rudd KE, Johnson SC, Agesa KM et al (2020) Global, regional, and national sepsis incidence and mortality, 1990–2017: analysis for the Global Burden of Disease Study. Lancet 395:200–211 31954465 10.1016/S0140-6736(19)32989-7 PMC6970225

[2] Singer M, Deutschman CS, Seymour CW et al (2016) The third international consensus definitions for sepsis and septic shock (Sepsis-3). JAMA 315:801 26903338 10.1001/jama.2016.0287 PMC4968574

[3] Barnett ML, Linder JA (2014) Antibiotic prescribing for adults with acute bronchitis in the United States, 1996–2010. JAMA 311:2020 24846041 10.1001/jama.2013.286141 PMC4529023

[4] Cassini A, Högberg LD, Plachouras D et al (2019) Attributable deaths and disability-adjusted life-years caused by infections with antibiotic-resistant bacteria in the EU and the European Economic Area in 2015: a population-level modelling analysis. Lancet Infect Dis 19:56–66 30409683 10.1016/S1473-3099(18)30605-4 PMC6300481

[5] Giacomini E, Perrone V, Alessandrini D et al (2021) Evidence of antibiotic resistance from population-based studies: a narrative review. Infect Drug Resist 14:849–858 33688220 10.2147/IDR.S289741 PMC7937387

[6] Tang BM, Eslick GD, Craig JC et al (2007) Accuracy of procalcitonin for sepsis diagnosis in critically ill patients: systematic review and meta-analysis. Lancet Infect Dis 7:210–217 17317602 10.1016/S1473-3099(07)70052-X

[7] Heffernan AJ, Denny KJ (2021) Host diagnostic biomarkers of infection in the ICU: where are we and where are we going? Curr Infect Dis Rep 23:4 33613126 10.1007/s11908-021-00747-0 PMC7880656

[8] Zimmermann T, Brealey D, Singer M (2024) Diagnosing sepsis: where we’re at and where we’re going. Intensive Care Med 50:957–959 38695925 10.1007/s00134-024-07428-1

[9] Barichello T, Generoso JS, Singer M et al (2022) Biomarkers for sepsis: more than just fever and leukocytosis—a narrative review. Crit Care 26:14 34991675 10.1186/s13054-021-03862-5 PMC8740483

[10] Larsen FF, Petersen JA (2017) Novel biomarkers for sepsis: a narrative review. Eur J Intern Med 45:46–50 28965741 10.1016/j.ejim.2017.09.030

[11] Pierrakos C, Velissaris D, Bisdorff M et al (2020) Biomarkers of sepsis: time for a reappraisal. Crit Care 24:287 32503670 10.1186/s13054-020-02993-5 PMC7273821

[12] Gant V, Singer M (2022) Combining pathogen and host metagenomics for a better sepsis diagnostic. Nat Microbiol 7:1713–1714 36289401 10.1038/s41564-022-01255-0

[13] Kalantar KL, Neyton L, Abdelghany M et al (2022) Integrated host-microbe plasma metagenomics for sepsis diagnosis in a prospective cohort of critically ill adults. Nat Microbiol 7:1805–1816 36266337 10.1038/s41564-022-01237-2 PMC9613463

[14] Stríz I, Trebichavský I (2004) Calprotectin–a pleiotropic molecule in acute and chronic inflammation. Physiol Res 53:245–253 15209531

[15] Lipcsey M, Hanslin K, Stålberg J et al (2019) The time course of calprotectin liberation from human neutrophil granulocytes after *Escherichia coli* and endotoxin challenge. Innate Immun 25:369–373 31109223 10.1177/1753425919848476 PMC7103615

[16] Wang S, Song R, Wang Z et al (2018) S100A8/A9 in inflammation. Front Immunol 9:1298 29942307 10.3389/fimmu.2018.01298 PMC6004386

[17] Kapel N, Ouni H, Benahmed NA et al (2023) Fecal calprotectin for the diagnosis and management of inflammatory bowel diseases. Clin Transl Gastroenterol. 10.14309/ctg.0000000000000617 37440723 10.14309/ctg.0000000000000617 PMC10522095

[18] Jukic A, Bakiri L, Wagner EF et al (2021) Calprotectin: from biomarker to biological function. Gut 70:1978–1988 34145045 10.1136/gutjnl-2021-324855 PMC8458070

[19] Gao R-Y, Jia H-M, Han Y-Z et al (2022) Calprotectin as a diagnostic marker for sepsis: a meta-analysis. Front Cell Infect Microbiol 12:1045636 36519133 10.3389/fcimb.2022.1045636 PMC9742445

[20] Zimmermann T, Brealey D, Singer M (2025) The search for sepsis biomarkers: a tale of promises, pitfalls, and potential. Crit Care Med 53:e543–e547 39692567 10.1097/CCM.0000000000006560

[21] Barnes J, Hunter J, Harris S et al (2019) Systematic review and consensus definitions for the Standardised Endpoints in Perioperative Medicine (StEP) initiative: infection and sepsis. Br J Anaesth 122:500–508 30857606 10.1016/j.bja.2019.01.009 PMC6435904

[22] Mylemans M, Nevejan L, Van Den Bremt S et al (2021) Circulating calprotectin as biomarker in neutrophil-related inflammation: pre-analytical recommendations and reference values according to sample type. Clin Chim Acta 517:149–155 33689693 10.1016/j.cca.2021.02.022

[23] Harris PA, Taylor R, Thielke R et al (2009) Research electronic data capture (REDCap)—a metadata-driven methodology and workflow process for providing translational research informatics support. J Biomed Inform 42:377–381 18929686 10.1016/j.jbi.2008.08.010 PMC2700030

[24] Lopez-Ayala P, Riley RD, Collins GS et al (2025) Dealing with continuous variables and modelling non-linear associations in healthcare data: practical guide. BMJ 390:e082440 40670054 10.1136/bmj-2024-082440

[25] R Core Team: R. (2024) A language and environment for statistical computing. Vienna, Austria: R Foundation for Statistical Computing. https://www.R-project.org/

[26] Christensen EE, Binde C, Leegaard M et al (2022) Diagnostic accuracy and added value of infection biomarkers in patients with possible sepsis in the emergency department. Shock 58:251–259 36130401 10.1097/SHK.0000000000001981 PMC9584040

[27] Havelka A, Sejersen K, Venge P et al (2020) Calprotectin, a new biomarker for diagnosis of acute respiratory infections. Sci Rep 10:4208 32144345 10.1038/s41598-020-61094-z PMC7060262

[28] Jonsson N, Nilsen T, Gille-Johnson P et al (2017) Calprotectin as an early biomarker of bacterial infections in critically ill patients: an exploratory cohort assessment. Crit Care Resusc 19:205–213 28866970

[29] Simm M, Söderberg E, Larsson A et al (2016) Performance of plasma calprotectin as a biomarker of early sepsis: a pilot study. Biomark Med 10:811–818 27414210 10.2217/bmm-2016-0032

[30] Diehl-Wiesenecker E, Galtung N, Dickescheid J et al (2024) Blood calprotectin as a biomarker for infection and sepsis–the prospective CASCADE trial. BMC Infect Dis 24:496 38755564 10.1186/s12879-024-09394-x PMC11100246

[31] Lengquist M, Sundén-Cullberg V, Hyllner S et al (2025) Calprotectin as a sepsis diagnostic marker in critical care: a retrospective observational study. Sci Rep 15:15529 40319081 10.1038/s41598-025-95420-0 PMC12049440

