# Supplementary material for: Evaluation of plasma calprotectin as a marker for infection in various clinical settings: a prospective observational study
Source: Intensive Care Med Exp. 2026 May 29;14:64. doi: 10.1186/s40635-026-00923-3 (PMC13219622; doi:10.1186/s40635-026-00923-3)
Supplement: Supplementary file 1 — Supplementary Material 1. [file 40635_2026_923_MOESM1_ESM.docx]

# *SUPPLEMENTARY MATERIAL*

# Evaluation of Plasma Calprotectin as a Marker for Infection in Various Clinical Settings: a prospective observational study

Tobias Zimmermann^1,2^; Timothy Arthur Chandos Snow^1^; Pedro Lopez-Ayala^3^; Ahmed Al-Hindawi^1^; Samer Elkhodair^4^; Nishkantha Arulkumaran^1^; Martin Siegemund^2^; Mervyn Singer^1,‡^; and David Brealey^1,‡^

^1^Bloomsbury Institute of Intensive Care Medicine, University College London, London, United Kingdom

^2^Intensive Care Unit, Department of Acute Medicine, University Hospital Basel, University of Basel, Switzerland

^3^Cardiovascular Research Institute Basel, University Hospital Basel, University of Basel, Basel, Switzerland.

^4^Emergency Department, University College London Hospital, London, United Kingdom

^‡^ Both authors contributed equally and should be considered last authors.

## Supplementary Methods

**Statistical analysis:**

Restricted cubic spline functions were used to model continuous non-linear associations between the biomarkers and outcomes, as recommended by Harrell.(1) Four knots were used for both Calprotectin and CRP and 3 knots for all other continuous variables.

## Supplementary Figures


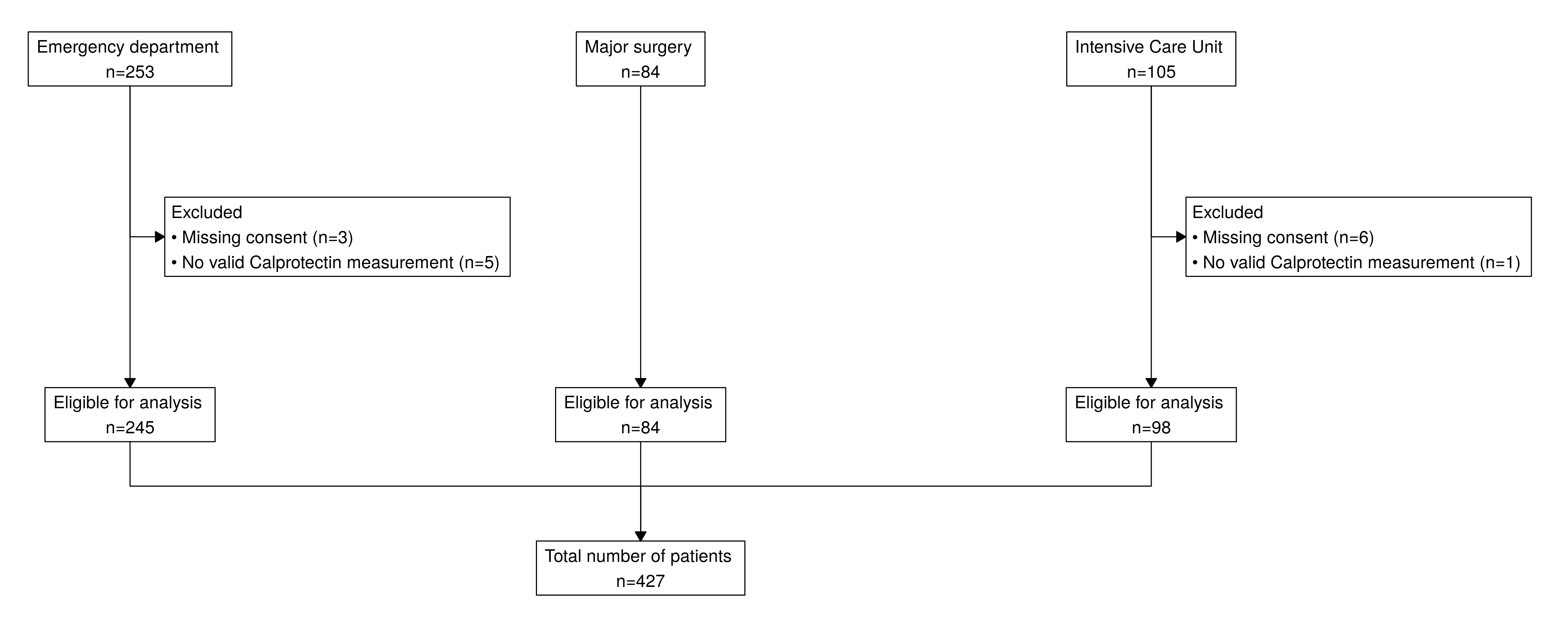


**Supplementary Figure 1**: Patient flowchart.


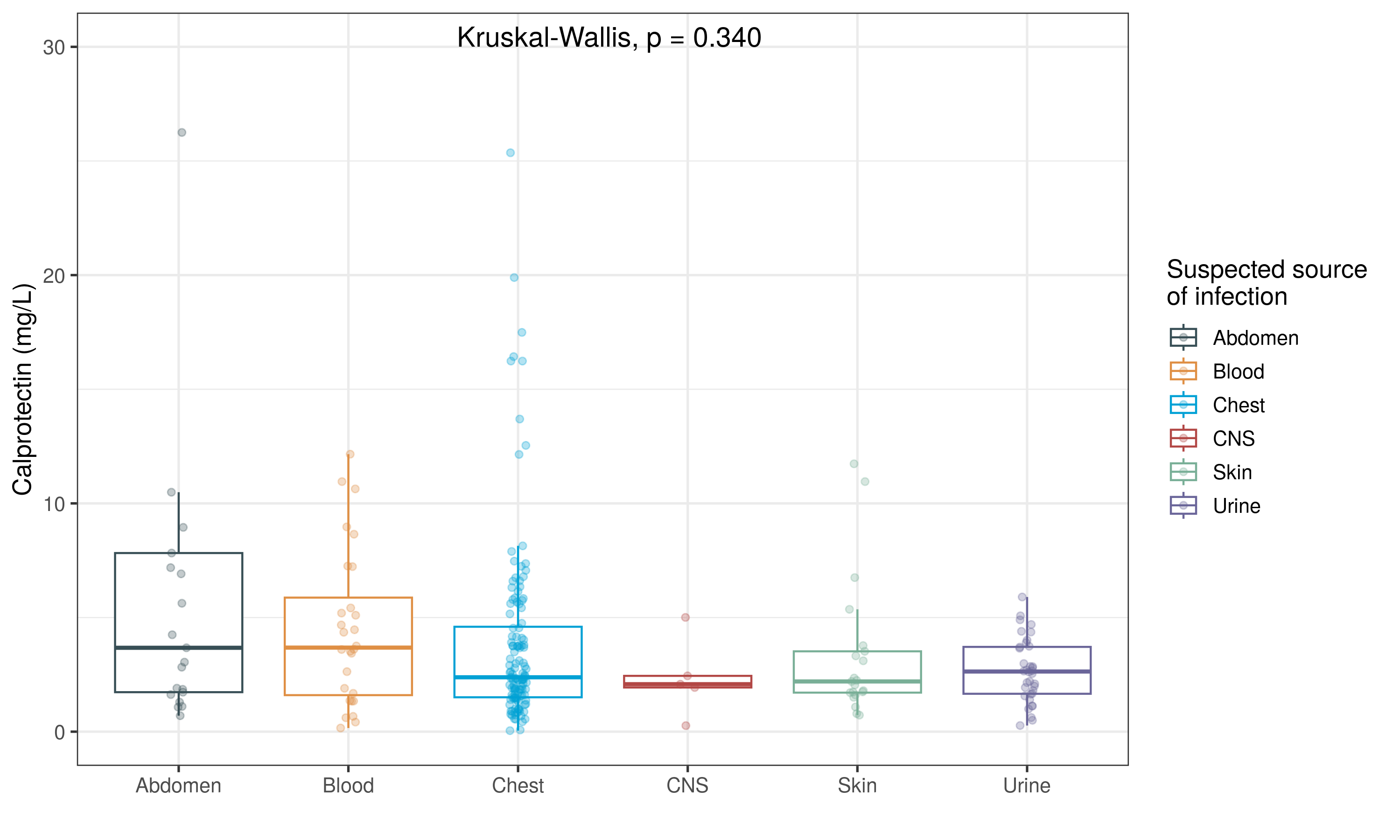


**Supplementary Figure 2**: Calprotectin concentrations of patients in the emergency department cohort, stratified by the suspected source of infection. CNS: central nervous system.


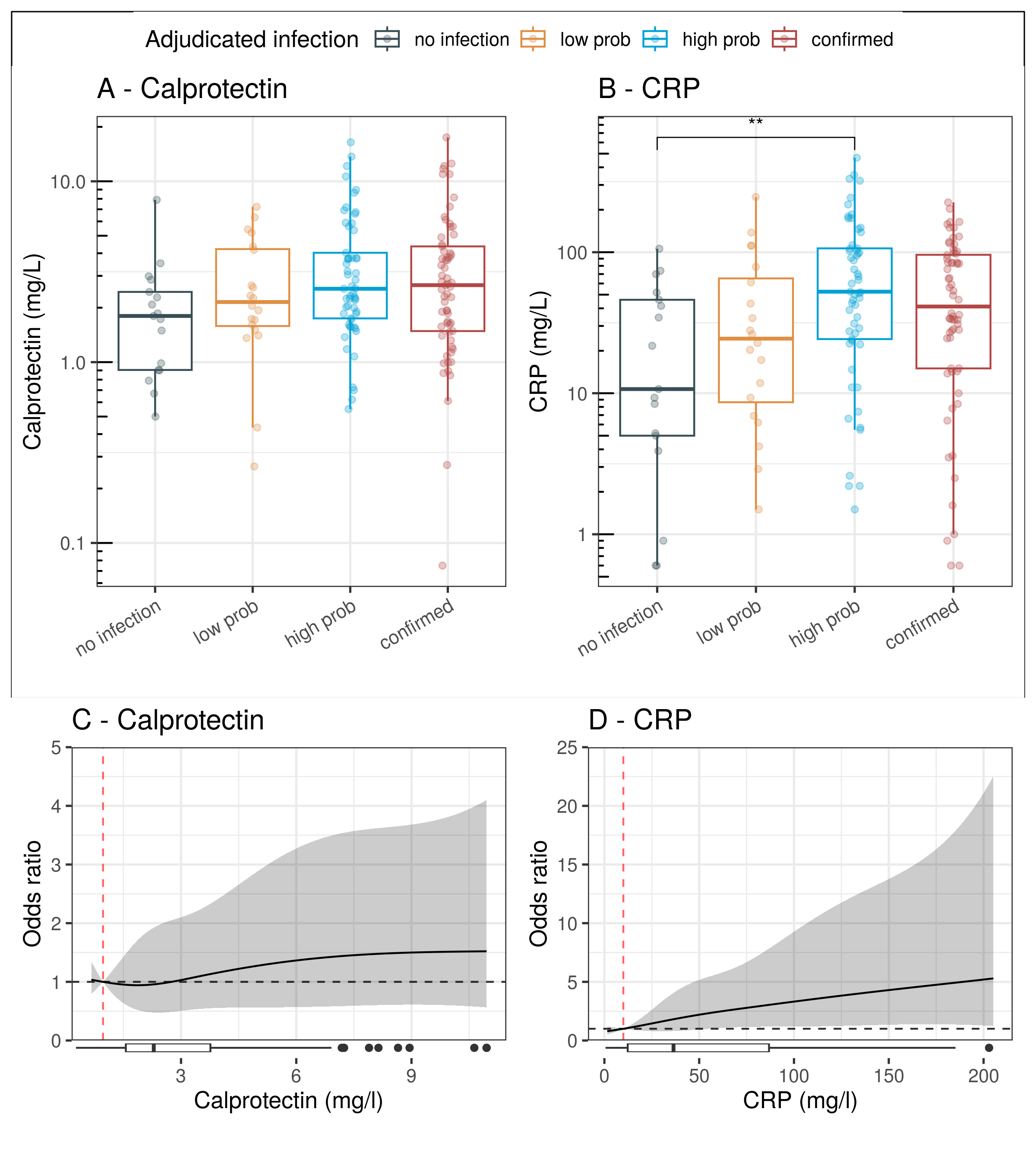


**Supplementary Figure 3**: Sensitivity analysis: Calprotectin (A) and CRP (B) concentrations on a logarithmic scale, in the emergency department cohort excluding patients with cancer and/or immunosuppression, stratified by the four adjudicated diagnoses: no infection, low probability, high probability, and confirmed infection. Only significant differences between groups are displayed. Dose-response odds ratio plots show the level-dependent association of Calprotectin (C) and CRP (D) with the outcome. The horizontal boxplot at the bottom displays the distribution of the biomarker data. Horizontal dashed black line: odds ratio of 1. Vertical red dashed line: reference levels of the biomarkers (Calprotectin 0.97mg/L, CRP 10mg/L). While dose-response plots were generated using all available data, only 95% of the data is displayed for better visualisation. prob: probability.


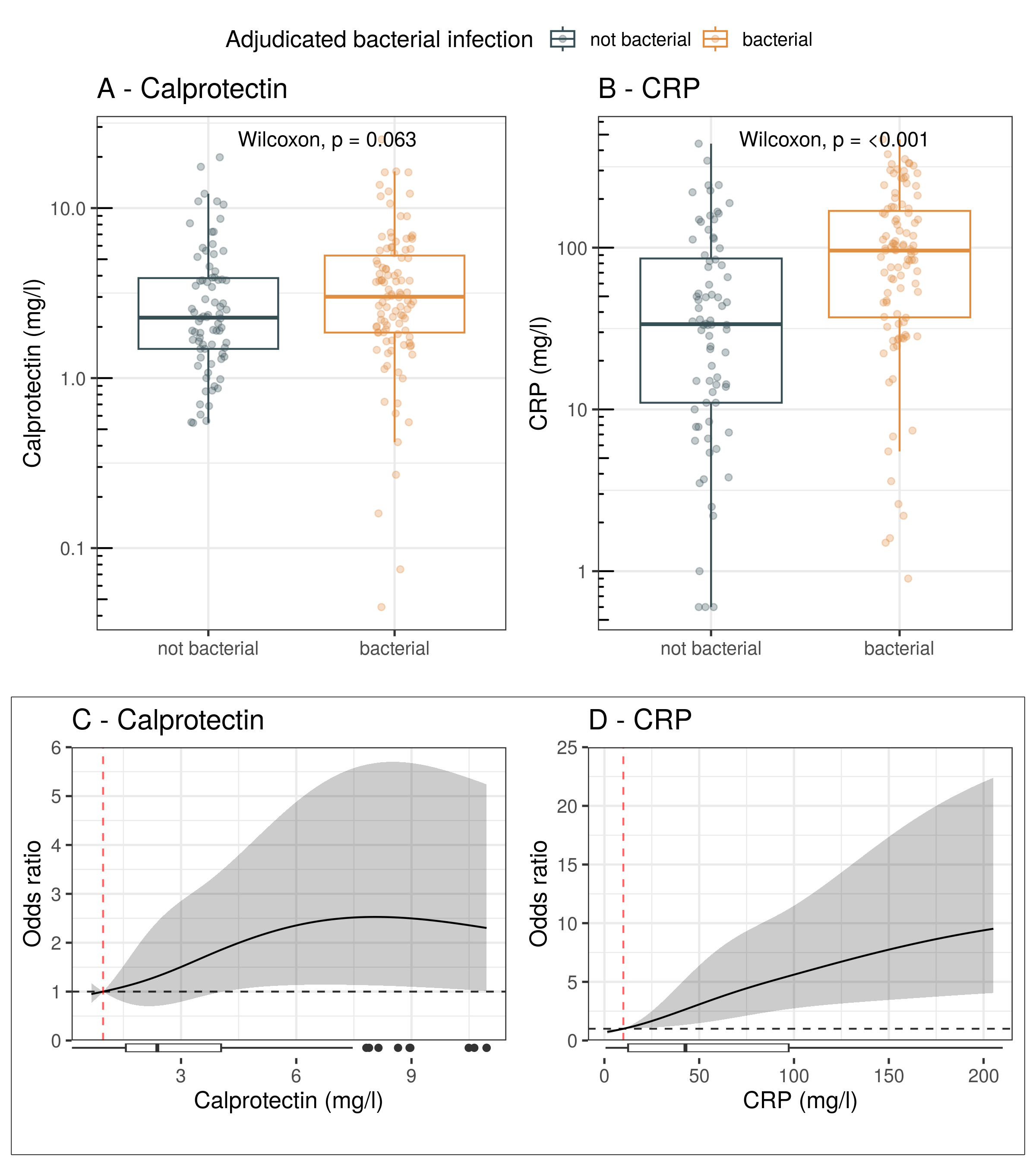


**Supplementary Figure 4**: Sensitivity analysis: Calprotectin (A) and CRP (B) concentrations on a logarithmic scale, in the emergency department cohort, stratified by an adjudicated diagnosis of bacterial infection. Dose-response odds ratio plots show the level-dependent association of Calprotectin (C) and CRP (D) with the outcome. The horizontal boxplot at the bottom displays the distribution of the biomarker data. Horizontal dashed black line: odds ratio of 1. Vertical red dashed line: reference levels of the biomarkers (Calprotectin 0.97mg/L, CRP 10mg/L). While dose-response plots were generated using all available data, only 95% of the data is displayed for better visualisation.


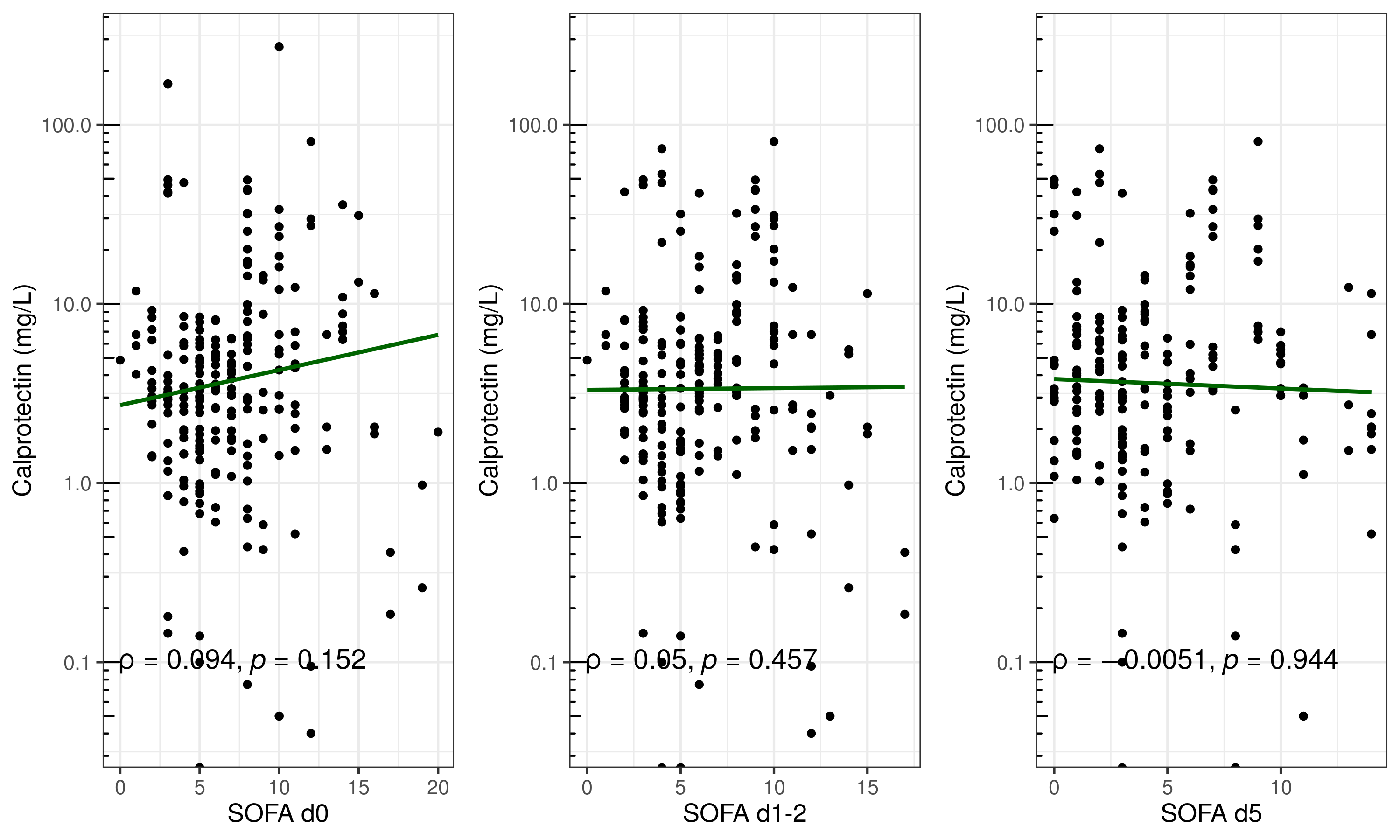


**Supplementary Figure 5**: Correlation analysis between Calprotectin and SOFA scores in the intensive care unit cohort at sampling time points day 0 (A), day 1-2 (B), and day 5-7 (C). Calprotectin concentrations (y-axis) are on a logarithmic scale.

## References

1. Harrell FE. Regression Modelling Strategies. 2nd ed. Springer; 2015.
